# Therapeutic Potential of White Kidney Beans (*Phaseolus vulgaris*) for Weight Management

**DOI:** 10.3390/foods14223940

**Published:** 2025-11-18

**Authors:** Hassan Muzaffar, Muhammad Jehangir, Jiayue Hu, Yiyang Yu, Mingzhou Yu, Yonghong Hu

**Affiliations:** 1 College of Food Science and Light Industry, Nanjing Tech University, Nanjing 211816, China; hassan14665@gmail.com (H.M.); yuyy@njtech.edu.cn (Y.Y.); 2 School of Food Science and Engineering, South China University of Technology, Guangzhou 510640, China; 3 Jiangsu Provincial University Key Laboratory of Intelligent Medical Sensing Materials and Devices, Institute of Chemical Biology and Functional Molecules, School of Chemistry and Molecular Engineering, Nanjing Tech University, Nanjing 211816, China; jehangirm62@gmail.com; 4 College of Biotechnology and Pharmaceutical Engineering, Nanjing Tech University, Nanjing 211816, China; 202361218090@njtech.edu.cn

**Keywords:** *Phaseolus vulgaris*, white kidney bean, anti-obesity, starch inhibition, fat metabolism, protein hydrolysates, alpha-amylase inhibitor (α-AI), functional food

## Abstract

The escalating global prevalence of obesity underscores the need for effective and sustainable nutritional interventions. Functional foods, especially white kidney beans (*Phaseolus vulgaris*), show a promising avenue to link fundamental biochemical insights with clinically feasible interventions, supporting their potential as an adjunct dietary strategy for managing and preventing obesity. This review critically examines the mechanistic roles of white kidney bean in weight regulation, which includes suppression of starch digestion, attenuation of postprandial glycemia, modulation of appetite and satiety, and hypolipidemic effects. Clinical and preclinical evidence supports the potential of white kidney bean as a nutraceutical for metabolic health, demonstrating consistent reductions in body fat mass, glycemic excursion, and overall weight. Nevertheless, significant limitations persist, including heterogeneity in trial designs, absence of dose standardization, and inadequate long-term safety assessments. Furthermore, this review addresses food fortification, advancements in supplement formulation, and cooking techniques that enhance both consumer acceptability and the bioactivity of white kidney bean (WKB), along with the significance of regulatory standards to ensure safety and quality. Future research should integrate clinical, molecular and food technology methods to improve the translation of experimental findings into precision nutritional strategies for obesity management.

## 1. Introduction

Obesity and overweight represent major contemporary health concerns. Obesity ranks as the fifth leading risk factor for global mortality [1]. According to the World Health Organization, over 0.83 billion individuals were affected by obesity in 2020, with projections indicating an increase to more than 1.53 billion by 2035 [2]. Obesity results from an energy imbalance between intake and expenditure, often attributable to insufficient physical activity and consumption of diets high in fats and sugars but low in fiber and micronutrients, such as vitamins and minerals [3].

Obesity is associated with several diseases, including type 2 diabetes, cardiovascular disease, and various cancers [4,5,6,7]. Irrespective of genetic predisposition, an unhealthy lifestyle combined with obesity can precipitate type 2 diabetes [8,9]. The primary strategies for managing excess weight encompass diet and physical activity [10]. Numerous dietary regimens and nutritional approaches featuring varying proportions of proteins, fats, and carbohydrates have been proposed for weight loss [11,12]. In recent years, restricting carbohydrate intake particularly processed carbohydrates, has emerged as an prominent weight loss strategy [13]. Given that carbohydrates constitute a primary caloric source in most diets, inhibiting or delaying their digestion is regarded as an effective strategy for weight management [14]. Recently, white kidney beans has garnered substantial interest for its capacity to inhibit carbohydrate digestion and facilitate weight management [15].

**Figure 1:**
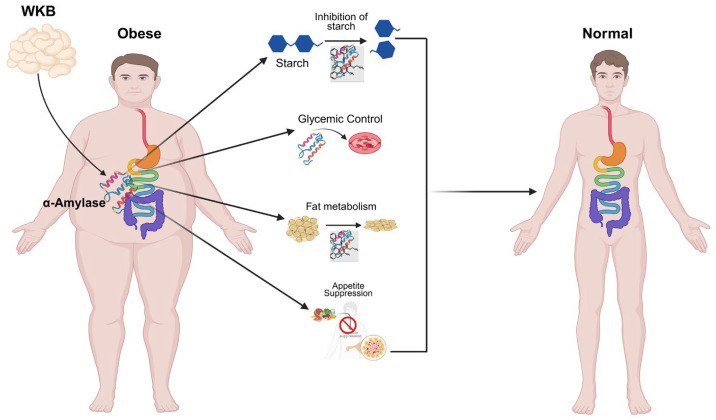
The Therapeutic Role of White Kidney Beans in Obesity Control.

White kidney beans (*Phaseolus vulgaris* L.) are native to the Mesoamerican and Andean regions [16]. They are abundant in complex carbohydrates, protein and bioactive compounds that promote metabolic health [3]. White kidney beans, which are rich in potassium (K) and magnesium (Mg), have been reported to confer benefits in individuals with conditions, such as atherosclerosis and hyperlipidemia [17]. *Phaseolamin*, a naturally occurring α-amylase inhibitor found in beans, impedes starch hydrolysis and retards carbohydrate digestion [18,19]. This process reduces caloric absorption, lowers the glycemic index of meals, and facilitates blood glucose regulation [20,21,22]. WKBE also enhances metabolic equilibrium and beneficially modulates the gut microbiota [23,24]. Furthermore, it mitigates fat accumulation and hyperglycemia while promoting weight loss [25]. A recent study indicates that α-amylase inhibitors derived from white kidney beans exhibit no adverse effects on the human body and can serve as safe dietary agents [26]. It also effectively regulates energy intake and appetite, thereby demonstrating promising outcomes in clinical studies involving obese patients, as illustrated in Figure 1 [27]. Although numerous studies have documented the nutritional properties and therapeutic importance of white kidney beans (*Phaseolus vulgaris*), a comprehensive synthesis of their impact on weight management remains scarce.

This review summarizes the current scientific literature on the pivotal role of white kidney bean extract for the prevention and treatment of obesity and related metabolic diseases. WKBE confers metabolic benefits extending beyond carbohydrates catabolism. Its elevated protein and fiber content promote satiety and appetite suppression. Studies in both animal and human models have demonstrated that WKBE improves lipid metabolism and reduces fat accumulation. Existing evidence from current and prior research supports WKBE as a valuable supplement for obesity and metabolic health management.

## 2. Methodology

### 2.1. Study Design

The manuscript presents a narrative review that provides a comprehensive critical synthesis and integration of recent scientific literature regarding the anti-obesity and metabolic effects of white kidney bean (*Phaseolus vulgaris*) extracts. The approach was chosen to organize information from diverse study models, including in vitro enzyme assays, phytochemical analysis and human clinical trials.

### 2.2. Literature Search Strategy

A systematic literature search was conducted across multiple electronic databases to encompass the contemporary scientific literature. Searches were conducted in key databases, including Google Scholar, PubMed, Xmol, Elict and Scispace with the final update in July 2025. The search strategy employed a combination of keywords, including “white kidney bean extract”, “*Phaseolus vulgaris*”, “phaseolamin”, “weight loss”, “weight management”, “obesity”, “alpha-amylase inhibitor”, “clinical trial”, “RCT”, “dose standardization”, “safety”, “consumption”, etc.

## 3. Nutritional Profile and Functional Properties of Pulses

Pulses exhibit a distinctive nutritional profile, featuring high protein content, low fat levels, and substantial dietary fiber. They are also abundant in complex carbohydrates, resistant starch, and essential vitamins, including folate (vitamin B9) [28]. Moreover, pulses contain a variety of non-nutritive bioactive compounds, including enzyme inhibitors, phytates, saponins, lectins, phenolic compounds, and oligosaccharides. These compounds contribute to their health-promoting effects and play a role in reducing the risk of non-communicable diseases linked to dietary and lifestyle changes [29]. Pulses serve as an excellent source of essential nutrients, comprising approximately 21–25% crude protein, 60–65% carbohydrates, and key micronutrients including magnesium, iron, potassium, and B vitamins [30]. They provide both soluble and insoluble dietary fiber, which contributes to weight management and cholesterol regulation. In addition, the bioactive compounds present in pulses exhibit anti-inflammatory and anticancer properties. Daily consumption of 100–200 g of pulses can fulfill recommended intakes of minerals and iron, rendering them a valuable element of a balanced diet [31]. Pulses represent a suitable and cost-effective source of protein, carbohydrates, vitamins, and minerals for obesity prevention, diabetes management, and chronic disease risk reduction [32]. They satisfy the increasing demand for dietary proteins, alleviate protein-energy malnutrition, improve soil fertility, and diminish greenhouse gas emissions [33]. Pulses are incorporated into traditional and innovative food products, including pasta, snacks, breakfast foods, and soups to enhance nutritional quality and technological attributes [34]. The gluten-free nature and abundance of resistant starch and fiber render them suitable for low-calorie and gluten-free diets. Techno-functional properties such as fat and water absorption capacity, foaming stability, and viscosity depend on their composition [35].

White kidney beans (*Phaseolus vulgaris* L.) are a widely consumed pulse and a vital global source of nutrients and dietary protein [36]. They display diverse physicochemical, cooking, hydration, and nutritional properties [37]. Although certain studies have investigated their functional properties, the digestibility and techno-functional attributes of diverse accessions remain largely unexamined [38].

White kidney beans are nutrient-dense, providing a balanced profile of complex carbohydrates, dietary fiber, and proteins. Their carbohydrate fraction, primarily comprising complex polysaccharides, facilitates slower digestion and absorption, thereby reducing postprandial glucose and insulin levels and potentially aiding weight loss. Dietary fiber supports gastrointestinal health and extends satiety, thereby contributing to weight management. Their high protein content and superior digestibility render white kidney beans a valuable staple in vegetarian and vegan diets [39].

## 4. Chemical Composition of White Kidney Beans

The chemical composition of white kidney beans (*Phaseolus vulgaris*) encompasses a broad spectrum of macronutrients and micronutrients, rendering them a valuable nutritional element [40]. These beans primarily consist of carbohydrates and proteins, with minor lipid content, along with essential minerals and amino acids [41].

### 4.1. Macronutrients

White kidney beans (*Phaseolus vulgaris*) are nutrient-rich legumes with a macronutrient profile predominantly comprising proteins and carbohydrates, accompanied by a minor fat fraction. This profile positions them as a staple plant-based functional food.

#### 4.1.1. Proteins

White kidney beans contain substantial protein levels, typically ranging from 20% to 33% of dry weight [42]. Globulins predominate, primarily phaseolin and vicilin, constituting 50–70% of total protein. Albumins comprise approximately 20% of the protein fraction, thereby augmenting nutritional quality [43]. These proteins are abundant in essential amino acids yet, akin to other legumes, relatively deficient in methionine. White kidney beans offer a favorable ratio of essential amino acids, ranging from 0.29% to 0.36%, underscoring their value as a plant-based protein source [44]. Processing methods, such as cooking and roasting, can enhance the protein content and enhance digestibility [45]. Moreover, isolated proteins from white kidney beans exhibits outstanding solubility and functional properties, rendering them suitable for diverse food applications. These isolated proteins interact with starch to form complexes that retard starch digestion, thereby lowering the glycemic index and promoting weight loss [46,47].

#### 4.1.2. Carbohydrates

Carbohydrates represent the predominant macronutrient in white kidney beans, constituting 45–60% of dry weight [48]. Starch is the primary carbohydrate, comprising 70.9–83.1% of the total, with a high proportion of resistant starch (RS) and slowly digestible starch (SDS) [49]. This composition suggests that an extensive portion of the starch is not directly broken down in the digestive tract, which helps with weight loss and regulates blood sugar levels after consumption [50]. They also contain a variety of non-starch polysaccharides, including mannose, galactose, arabinose and galacturonic acid. White kidney beans exhibit low sugar content, rendering them appropriate for low-sugar diets [51].

#### 4.1.3. Lipids

White kidney bean (WKB) contains minimal fat, ranging from 1% to 2.8%. The majority comprises polyunsaturated fatty acids, particularly linolenic and α-linolenic acids, which support cardiovascular health. Their low-fat profile renders white kidney beans ideal for weight management and cardiovascular disease [43,44].

### 4.2. Micronutrients

White kidney beans (*Phaseolus vulgaris*) contain a variety of micronutrients, including essential minerals and vitamins, which underscore their nutritional and health-promoting attributes [17].

#### 4.2.1. Minerals

White kidney beans are rich in calcium, magnesium, potassium, phosphorus and zinc. For example, the average amounts recorded for kidney beans are approximately 8.2 mg iron, 407 mg phosphorus, and 143 mg calcium per 100 g of dry weight, with substantial potassium and magnesium content [52]. These minerals play an important role in various physiological activities, including bone health (phosphorus and calcium), oxygen transport (iron), and potassium and magnesium for muscle function and the nervous system, as well as zinc for immune support [53]. The mineral content can be modulated through agronomic practices; for example, biofortification with zinc sulfate and iron has been reported to elevate levels in kidney beans, thereby enhancing nutritional value [54]. Moreover, application of natural fertilizers, such as zeolite and sheep manure can increase concentrations of potassium, iron, and zinc [55].

#### 4.2.2. Vitamins

White kidney beans are a suitable source of various types of B vitamins. Including B1 thiamine, B2 riboflavin, B3 niacin, and B6 pyridoxine, which are necessary for the central nervous system and energy metabolism [56]. They also contain vitamin E (predominantly γ-tocopherol), a fat-soluble antioxidant that assists in protecting cells from damage caused by oxidative stress. Moreover, they are not rich in fat-soluble vitamins such as vitamins A, D, K, and C like other legumes [43].

#### 4.2.3. Bioactive Compounds

**Figure 2:**
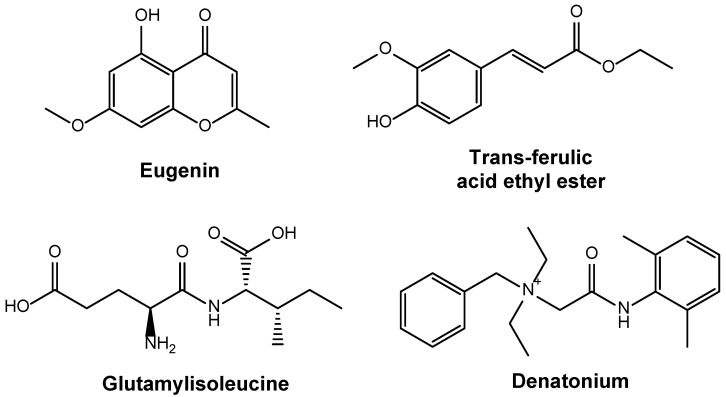
Representative bioactive compounds identified in *Phaseolus vulgaris*.

White kidney beans (*Phaseolus vulgaris*) contain large amounts of flavonoids and phenolic compounds. The overall quantity of flavonoid content ranges from 0.19 to 7.05 mg/g, while the phenolic compounds vary from 0.25 to 0.37 mg rutin equivalent (RE)/g dry weight. These chemical compounds can exhibit antioxidant activities [57]. Recent studies haved identified various distinct bioactive compounds in *Phaseolus vulgaris*, such as eugenin, trans-ferulic acid ethyl ester, glutamyl isoleucine α-L-glutamyl-L-leucine and denatonium as shown in Figure 2; however, their precise proportions remain undetermined [58]. Isolation and characterization of these bioactive compounds from white kidney beans confirm the presence of diverse compounds with multiple health benefits [59]. Relative to flavonoids and phenolic compounds, white kidney beans contain lesser amounts of tannins, saponins and coumarins, which show antifungal and antibacterial properties [60].

## 5. Mechanism of Weight Loss

White kidney beans (*Phaseolus vulgaris*) have attracted considerable interest not only for their culinary versatility but also for their potential in weight management. A meta-analysis by Davkova et al. (2024) demonstrated that *Phaseolus vulgaris* supplementation exerts a significant effect on body weight and fat mass [61]. Upon ingestion, carbohydrates must be hydrolyzed and converted into monosaccharides for intestinal absorption [50]. The enzymes responsible for converting complex carbohydrates into monosaccharides are amylases and β-glucosidases. These enzymes are indispensable for this process [62]. Initially, amylase hydrolyzes complex starch into oligosaccharides. Subsequently, β-glucosidases further hydrolyze oligosaccharides into monosaccharides [63]. Studies have identified three α-amylase inhibitors in white kidney beans: α-AIL, α-A1, and α-A12. These inhibitors effectively attenuate amylase activity, thereby facilitating weight reduction [64].

### 5.1. α-Amylase Inhibition and Glycemic Control

**Figure 3:**
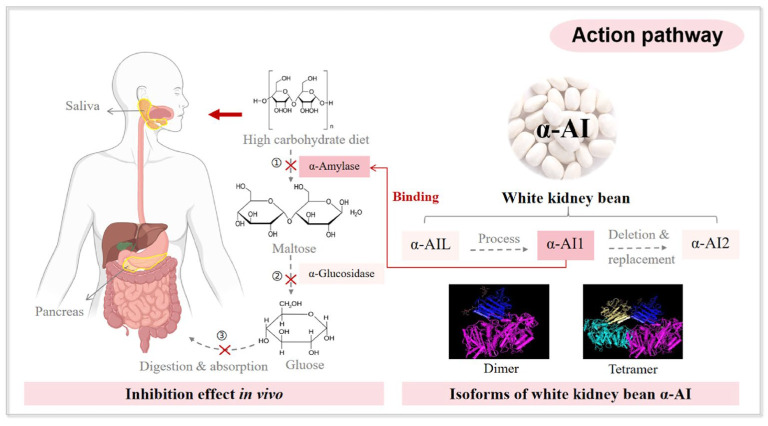
Insight into α-Amylase inhibition and glycemic control mechanisms of WKB. Reproduced with permission [66], Copyright 2024, Elsevier Ltd.

Inhibition of carbohydrate digestion represents a primary mechanism by which white kidney bean extract (WKBE) facilitates weight management. This is primarily attributable to its α-amylase inhibitory activity. This activity attenuates the hydrolysis of complex starches into absorbable monosaccharides. Consequently, carbohydrate absorption is delayed, resulting in attenuated postprandial glucose responses and reduced caloric intake, as illustrated in Figure 3 [65,66]. Preclinical studies in animal models corroborate this mechanism. Rodents administered WKBE exhibited reduced carbohydrate absorption, attenuated postprandial glycemia, and decreased body weight [23,67]. Long-term supplementation in animals also yielded reductions in visceral fat mass and improvements in metabolic flexibility, further substantiating the association between inhibited carbohydrate metabolism and body weight regulation [68]. Beyond inhibiting carbohydrate digestion, WKBE promotes weight management by stabilizing postprandial blood glucose and insulin levels, thereby improving overall glycemic control. This is particularly pertinent for individuals with metabolic disorders, such as diabetes [69,70,71]. Notably, in vitro gut model studies demonstrated α-amylase inhibition, whereas in vivo experiments failed to confirm significant enzyme inhibition [65]. This suggests that direct active-site inhibition may not represent the sole mechanism of α-amylase inhibition. Alternatively, WKBE may exert indirect effects, such as delayed starch hydrolysis, partial enzyme inhibition, or modulation of gut microbiota activity.

Overall, these findings indicate that WKBE exerts anti-obesity effects by diminishing carbohydrate absorption—via α-amylase inhibition and glycemic control—through modulation of gut microbiota and enzyme activity.

### 5.2. Gut Microbiota and Gastrointestinal Effects

**Figure 4:**
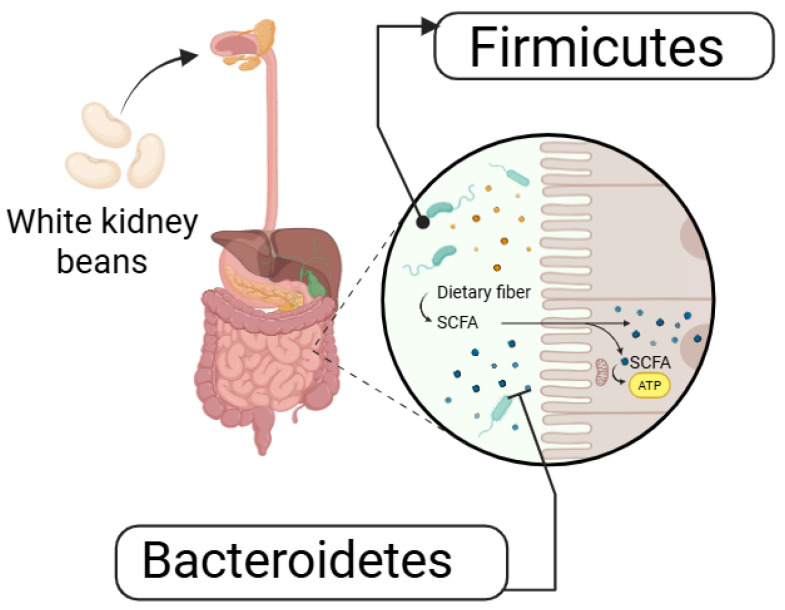
Gut Microbiota regulation by WKB.

WKBE can also alter gut microbiota composition. In a randomized, placebo-controlled crossover trial, Houghton et al. (2023) [72] assessed gut microbiota composition, gastrointestinal inflammation, and stool characteristics in 20 healthy individuals following seven days of white kidney bean (WKB) consumption. WKBE administration elevated the relative abundance of Firmicutes while reducing that of Bacteroidetes as shown in Figure 4 [72]. However, no significant differences were observed in digestive symptoms, stool frequency, or fecal calprotectin between the placebo and WKBE groups, indicating minimal effects on gastrointestinal health [73].

### 5.3. Appetite Suppression and Satiety

Alongside its effects on blood sugar regulation and carbohydrate metabolism, WKBE may also play a role in body weight management by controlling satiety and overall energy intake. This effect is partially attributable to its elevated protein and fiber content, which delay gastric emptying and promote satiety [74,75]. Moreover, α-amylase inhibition prolongs the release of glucose from starch, thereby stabilizing blood glucose levels and mitigating appetite fluctuations [76].

This satiety-promoting effect is corroborated by both human and animal studies. In rodents, supplementation with WKBE reduced food intake and body weight gain relative to controls. These results were linked to reduced ghrelin (the hunger hormone) and elevated peptide YY and GLP-1, hormones that regulate satiety [77]. These findings suggest that WKBE influences both behavioral and physiological dimensions of appetite, thereby supporting weight loss through diminished energy intake [78].

### 5.4. Fat Metabolism and Lipid Profile

An additional mechanism by which WKBE supports weight management involves improving lipid profiles and modulating fat metabolism. WKB contains bioactive compounds that may reduce lipid absorption in the intestine and promote fat utilization as an energy source [79]. Studies have shown that white kidney beans contain compounds capable of modulating lipid absorption and ameliorating lipid profiles. This is critical for both weight loss and cardiovascular health [64]. WKBE facilitates weight management through enhanced lipid profiles and modulated fat metabolism. WKB harbors bioactive compounds that may attenuate intestinal lipid absorption and enhance fat oxidation as an energy substrate [80]. This mechanism is pertinent to both weight loss and cardiovascular health.

Preclinical studies provide further mechanistic insights. Rodents fed a high-fat diet supplemented with WKBE demonstrated lower LDL cholesterol, reduced serum triglycerides and improved hepatic lipid metabolism. Histological investigations also exhibited reduced lipid accumulation in the adipose tissue and liver, consistent with decreased fat storage [81]. These studies confirm that WKBE reduces lipid absorption, enhances lipid catabolism, and mitigating fat deposition.

Both human and preclinical studies provide evidence of WKBE’s role in ameliorating lipid profiles, modulating lipid metabolism, and contributing to its overall anti-obesity effects [82].

## 6. Clinical Efficacy and Evidence Synthesis

Clinical trials have demonstrated that white kidney bean extract (WKBE) attenuates postprandial blood glucose levels and facilitates weight management. For instance, Barrett et al. (2011) [83] examined four crossover trials as shown in Table 1. These trials revealed that WKBE significantly reduced the postprandial glucose area under the curve (AUC) by up to 66% and accelerated return to baseline glucose levels, indicative of diminished carbohydrate absorption. A dose–response relationship was evident, with 1500 mg proving more efficacious than 750 mg, and a 3000 mg powdered formulation reducing postprandial hyperglycemia by 34.1% [84]. Furthermore, a multi-ingredient formulation containing 500 mg of the extract mitigated postprandial glucose and insulin excursions in individuals with elevated fasting blood glucose. Multiple studies confirm that WKBE supplementation promotes weight loss. Over periods of 35–84 days, participants receiving WKBE exhibited significantly greater weight loss (1.9–3.2 kg), along with reductions in waist circumference and body fat, relative to placebo groups [74,83,85,86]. This effect is attributable to enhanced satiety and diminished appetite, resulting in improved portion control and reduced food intake [70,87]. In addition to promoting weight loss, WKBE supplementation improves cardiovascular risk markers by lowering triglyceride and cholesterol levels while increasing HDL cholesterol [70]. These findings suggest that WKBE may concurrently facilitate weight loss and mitigate cardiovascular risk.

## 7. Transformative Processing and Bioactivity Enhancement

White kidney beans are abundant in proteins, bioactive compounds and phenolics; however, their limited bioavailability and antinutritional factors may compromise functional and nutritional benefits. The digestibility, bioactivity, and health benefits of white kidney beans can be enhanced through transformative processing techniques, ranging from conventional boiling to advanced technologies.

### 7.1. Enzymatic Hydrolysis

**Figure 5:**
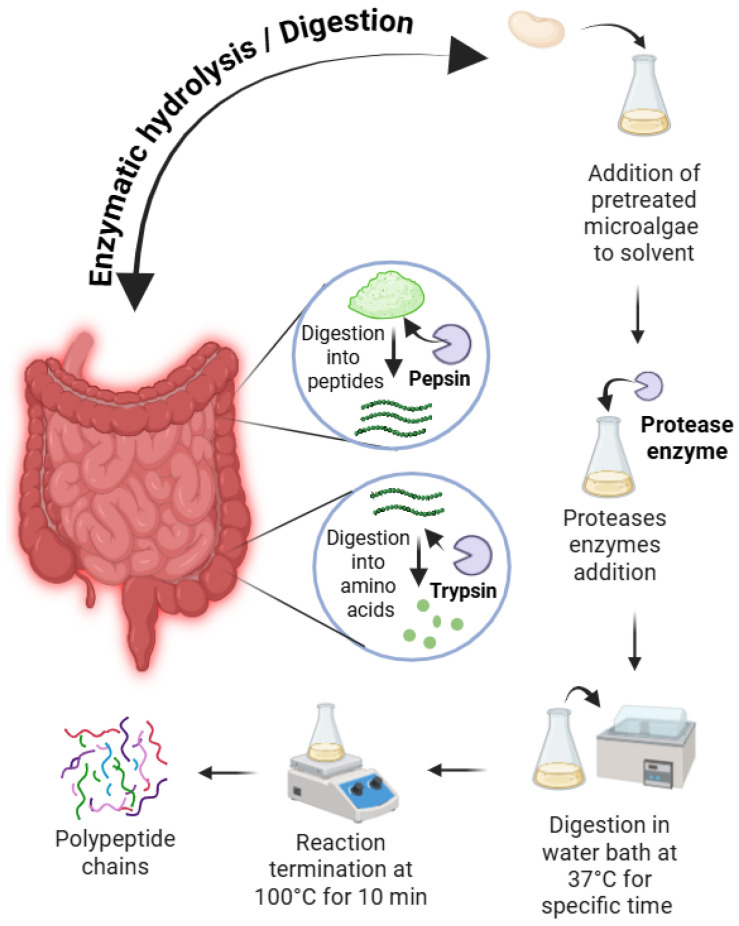
Enzymatic and chemical hydrolysis of WKB.

Protein fractions from white kidney beans are typically modified via enzymatic and chemical hydrolysis to augment their bioactive and functional properties for food and nutraceutical applications [90]. Enzymatic hydrolysis employs common proteases, such as pepsin, papain, alcalase, and flavourzyme as shown in Figure 5. These enzymes cleave peptide bonds, yielding protein hydrolysates that improve foaming, emulsifying, and solubility properties [91,92,93]. For instance, pepsin hydrolysis achieved a degree of hydrolysis (DH) of 8.6% (60 min), thereby enhancing solubility, emulsifying, and foaming properties [94]. Enzymatic hydrolysis generates bioactive peptides exhibiting antioxidant, antimicrobial, and antihypertensive activities. Furthermore, papain- or alcalase-derived hydrolysates display pronounced antioxidant and antibacterial properties and inhibit enzymes associated with diabetes and hypertension [95].

### 7.2. Chemical Hydrolysis

Chemical hydrolysis produces smaller peptides and amino acids from proteins using acids or bases. Although essential for complete protein degradation and amino acid analysis, chemical hydrolysis lacks the selectivity of enzymatic methods [96]. Proteins extracted from white kidney beans using citric acid and sodium hydroxide can subsequently be fractionated and analyzed; these methods alter protein fractions, charge properties, and solubility, thereby influencing functional characteristics [97]. Nevertheless, acid hydrolysis remains a standard method for determining amino acid composition in white kidney bean proteins, as it completely degrades proteins into constituent amino acids for analysis [96]. Chemical hydrolysis, however, has limitations; it can destroy certain amino acids, such as tryptophan, and generate undesirable byproducts, thereby reducing its suitability for producing food-grade protein hydrolysates [98]. Although efficient for analytical purposes, chemical hydrolysis is infrequently employed for producing bioactive peptides in nutraceutical applications owing to its lack of specificity [99].

### 7.3. Protein Functionalization and Modification Techniques

Techniques such as heat treatment, ultrasound, and pH-shifting can effectively modify the surface properties of white kidney bean protein concentrates. These modifications significantly enhance foaming capacity, solubility, and surface hydrophobicity—all critical for applications in baked goods and emulsified foods. Heat treatment and pH-shifting can improve foaming capacity while yielding the highest solubility and emulsifying stability [100]. Moreover, solid-state fermentation can enhance functional properties (emulsifying, foaming, and oil- and water-holding capacities) and mineral bioavailability, thereby promoting the incorporation of kidney bean flour into fortified foods [101].

### 7.4. Applications for Ingredient Formulation

Incorporating modified white kidney bean components into baked goods, pasta, and meat products fortifies these items, enhancing nutritional quality and functional properties, including texture, stability, and yield [92]. Employing chemical, enzymatic, physical, and fermentation methods functionalizes and modifies white kidney beans, significantly enhancing their value as food ingredients. These methods augment bioactivity, functional attributes, and nutritional value, rendering them suitable for diverse innovative and health-promoting food formulations [102].

## 8. Functional Aspects, Processing, and Consumer Aspects of WKB

WKB exhibits substantial chemical and nutritional properties, including abundant phaseolin protein, which comprises 20–30% of its content [42,103]. These beans are low in fat, with a high proportion of unsaturated fatty acids, appealing to consumers with cardiovascular disorders. Additionally, fiber and antioxidant compounds support digestive health in patients with chronic conditions [104]. The high resistant starch content in WKB contributes to a low glycemic index, rendering it promising for patients with hyperglycemia, which often precipitates overweight [105]. These nutritional and functional attributes are significantly influenced by WKB processing and extraction techniques.

### 8.1. Correlation Between Processing, Functional Aspects, and Consumer Acceptability of WKB

Biological processes substantially affect the nutritional profile of WKB. For instance, germination, fermentation, soaking, cooking, and autoclaving enhance nutrient bioavailability and digestibility while diminishing antinutritional factors [30,43,104]. Conversely, extraction and modification methods—including physical, chemical, and biological approaches—are applied to improve the solubility and emulsifying properties of WKB [90,103]. These attributes broaden their applicability as functional foods for consumers.

### 8.2. Correlation Between Functional and Consumer Aspects

WKB, with its substantial bioactive potential, is utilized to develop low-glycemic-index foods owing to its α-amylase inhibitor, which regulates blood glucose levels and mitigates overweight, particularly in diabetic patients [66]. The protein and fiber contents of WKB render it promising for plant-based products, such as meat analogs and dairy substitutes [106]. A primary consumer consideration is economic acceptability, as WKB serves as an inexpensive and sustainable protein source. This supports food security and yields economic benefits, particularly in developing regions [104].

## 9. Comparative Analysis of Other Protein Sources

### 9.1. Comparison with Animal-Based Proteins

Plant-derived proteins are increasingly replacing animal proteins in the United States. The market for plant-based meat has rapidly grown, and it is estimated to increase at a rate of 19.3% yearly between 2022 and 2030 [107]. The overall risk of mortality is directly related to red meat (beef) and, more specifically, to processed meat [108]. One potential reason for the observed association between beef consumption and a reduced risk of chronic illness is the high content of saturated fats, carcinogens generated during cooking, as well as heme iron-derived molecules, such as heterocyclic amines and N-nitroso compounds [45]. Heterocyclic amines (HAAS/HCAS) are generated when high-protein diets like red meat are cooked at high temperatures. These chemical compounds are classified as strong carcinogens and mutagens that can cause oxidative stress, inflammation, and DNA adduct formation. They may adversely damage DNA, disrupt normal cellular function and enhance the promotion of tumor growth in a number of organs, including the breast, liver, colon and others. Besides these effects, certain HAAS/HCAS) to protein aggregation and mitochondrial dysfunction, which can lead to neurodegenerative diseases such as Alzheimer’s and Parkinson’s diseases [109]. HCAs possess the ability to disrupt lipid metabolism, and cause renal and intestinal dysfunction, which may contribute to liver illnesses. N-nitroso compounds (NOCs), such as nitrosamines, are created endogenously in the stomach from dietary precursors during food processing. NOCs are extremely carcinogenic, genotoxic and mutagenic. In animal model studies, more than 90% of research indicated that NOCs are highly carcinogenic, leading to benign tumors in the gastrointestinal system. Diets focused mainly on plants have been linked to improved health [110]. Most beans and pulses are rich in resistant starch, dietary fiber, and essential micronutrients (e.g., iron, magnesium, copper, phosphorus, potassium, manganese, and B vitamins) [111]. Furthermore, they have a notable protein content of 21–25% by weight. The commonly consumed dry beans, Phaseolus vulgaris, have an average of 0.5 g of total fat per half-cup.

According to projections, common beans or Phaseolus vulgaris are a staple food for 500 million people due to their protein richness (22–27% of seed weight) and carbohydrates (39–47% of seed weight). A 100 g serving of dry common beans can provide 9–25 g of protein, meeting over 20% of an average adult’s daily requirement. The protein from beans meets the basic human protein needs as recommended by the FAO and WHO. Furthermore, several studies have shown the anti-inflammatory, antioxidant, anti-obesity, anti-carcinogenic, anti-diabetic, and cardioprotective potential of Phaseolus vulgaris extract (PVE) [112].

### 9.2. Comparison with Other Plant-Based Proteins

WKBs serve as an inexpensive source of proteins, vitamins, resistant starch, minerals, polyphenols, and dietary fiber. Legumes, when combined with cereal, typically provide a good source of protein (20–40%) as shown in Table 2 [113]. High protein content, widespread acceptance, and affordability of beans render them the most suitable source for protein isolate production. Among the most extensively grown and eaten legumes worldwide are beans and peas [114]. Peas (*Pisum sativum* L.) are the second-highest leguminous crop farmed on more than 25 million acres globally. Field peas contain higher levels of lysine and tryptophan than cereal grains and have lower trypsin inhibitor content than soybeans. According to Sai. UT et al. (2009) [115], navy beans exhibited the highest levels of ash, fat, and crude fiber, while red kidney beans showed the highest carbohydrate content. Adzuki bean showed the most vigorous trypsin-inhibitory activity. Globulin polypeptides were the predominant polypeptides in both the entire protein and the protein isolate, with molecular weights of 45–55 kDa. Every seed’s protein isolate contained at least one major glycopeptide, and the whole protein contained more than two. At pH 4–5, all protein extracts show minimal protein solubility. Regarding functional properties, kidney and navy beans demonstrated superior foamability and foam stability compared to adzuki beans. Adzuki beans exhibited the highest oil absorption capacity, whereas no significant difference in water absorption was observed between adzuki and kidney beans as shown in Table 2.

**Table 2:** Comparison of white kidney with plant and animal-based protein sources.

Category	Protein Source	Nutritional Composition	Health Benefits	Limitations	Ref
Legume Protein	White kidney bean	*Phaseolus vulgaris* contains around 22–27% protein. It also contains starch resistance, dietary fiber, minerals (K, Mg, Fe), and bioactive compounds like polyphenols, saponins, and α-amylase inhibitors.	WKB shows its potential role for anti-obesity, anti-diabetic, anti-inflammatory, antioxidant, cardio-protective, and hypolipidemic effects.	WKB contains some anti-nutritional components such as phytates, lectins, and trypsin inhibitors when insufficiently processed.	[116,117,118]
Animal-based Proteins	Fish, Beef, and dietary	It contains Protein, critical amino acids, heme iron, and B12.	It supplies essential nutrients, improves muscle growth, and whey and casein enhance satiety and lean mass.	It shows some serious adverse effects on human health. Red meat is connected to an increased cardiovascular risk and cancer, due to its high saturated fat content.	[119,120]
Plant-based Protein	Soy Protein	It includes 40% which is rich in essential amino acids and isoflavones.	It reduces cholesterol levels. Improves bone strength, cardio-protective, and helps weight management.	It contains phytoestrogen, the causative factor of allergy.	[121,122]
	Pea Protein (*Pisum sativum* L.)	It contains 20–25% protein, rich in tryptophan and lysine, and is less efficient than a trypsin inhibitor.	It enhances blood pressure and controls glucose level, boosts muscle protein synthesis, and increases satiety.	Pea protein shows low digestibility as compared to animal-based protein and low methionine.	[123,124,125]
	Other Legumes, including (Red Kidney, Navy, and adzuki Beans	Red kidney beans contain a high amount of carbohydrates, while navy beans have more fiber, and adzuki beans show strong trypsin-inhibitory activity.	Exhibits anti-carcinogenic properties, antioxidants, anti-hypertensive, and supports gastrointestinal health.	With the presence of tannins and trypsin inhibitors, their protein solubility is a challenge.	[125,126,127,128]

## 10. Safety and Side Effects

### 10.1. Allergenic Potential

The utilization of raw or inadequately processed kidney beans is constrained by antinutritional factors, stemming from various non-nutritional compounds that can interfere with normal metabolism, inhibit nutrient digestion and absorption, and initiate allergic reactions [129]. Consumption of raw or undercooked kidney beans can induce acute gastrointestinal symptoms attributable to phytohemagglutinin; this risk is mitigated by adequate heat treatment [130]. Bento et al. (2021) [131] highlighted that antinutritional factors—a diverse array of substances also known as non-nutritional factors or plant bioactive components, exert protective effects against various malignancies and coronary atherosclerotic disease. Antinutritional components, including toxic alkaloids and proteins, adversely affect animal and human health by reducing nutrient absorption from kidney beans (KBs) and their products [131].

**Figure 6:**
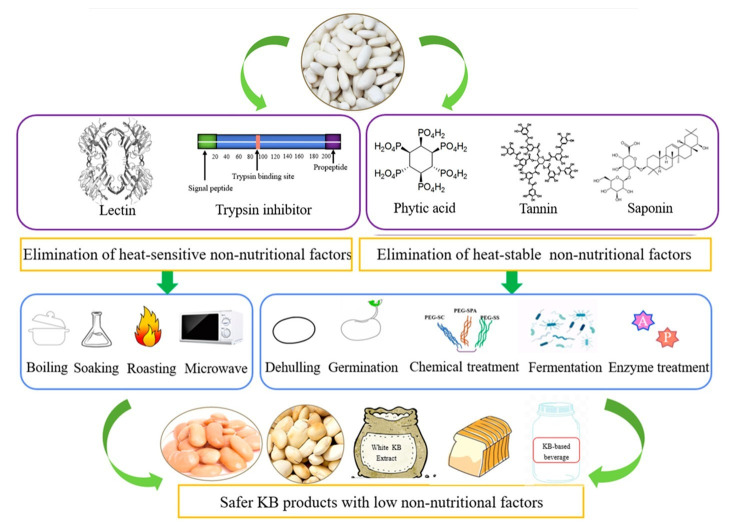
Methods for inactivating non-nutritional components in kidney beans, adopted with permission from [129].

Most antinutritional constituents in KBs comprise lectin, trypsin inhibitor, saponin, phytic acid, and other compounds capable of chelating ionic cofactors, directly inhibiting protease action, or forming irreversible complexes that impede protein digestion [95]. Methods for inactivating non-nutritional components in KBs and producing KB products with negligible levels of these constituents are shown in Figure 6 [129]. The non-nutritional components included in KBs are diverse in size and form, and each has its own physiological impact. Phytates and tannins bind proteins and minerals, thereby reducing their bioavailability and digestibility. Digestion of protein and starch is impacted by trypsin inhibitors, which bind proteases and diminish their activity. Lectins trigger allergic responses by binding to carbohydrate moieties on cell surfaces. These antinutritional factors can impair nutrient absorption in humans and animals, elicit allergic reactions, disrupt normal metabolism, and adversely affect the skin, respiratory, and gastrointestinal systems; in severe cases, they may cause food poisoning. Consequently, their presence restricts human growth and KB consumption by compromising the quality and nutritional value of KB products [132].

### 10.2. Digestive Issues

The α-amylase inhibitor inhibits starch digestion by blocking access to the enzyme’s active site. The inhibitory efficiency of α-AI isoforms is influenced by multiple parameters, including temperature, incubation duration, pH, and the presence of specific ions. The optimal temperature range is 22–37 °C, with an optimum pH of 4.5–5.5 for the inhibitor. Boiling beans for five minutes completely inactivate the inhibitor, while at 0 °C, its activity ceases. According to three separate studies, optimal incubation periods are 10, 40, and 100 min, varying test conditions—a pH of 4.5 for the shorter incubation periods and a pH of 6.9 for the longer ones, presumed to be the reason for the variations in incubation lengths [133]. Due to lectins and other antinutritional factors, consuming raw or inadequately processed KBs can decrease protein digestibility, impair the utilization and absorption of other nutrients, cause symptoms such as weight loss, loss of appetite, dyspnea, and, in severe cases, poisoning.

Trypsin inhibitors, known as anti-metabolic proteins, bind to digestive enzymes such as trypsin and chymotrypsin, blocking protein hydrolysis and the absorption of amino acids [134]. Tannins, water-soluble phenolic metabolites, are divided into three groups called hydrolysable tannins (as ellagitannins and gallotannins), complex tannins (like proanthocyanidins), and condensed tannins. Condensed tannins can precipitate and bind protein fractions, reducing their availability and making them hard to absorb and digest [135]. Saponins, known as tri-terpenoids or steroidal glycosides, present in KBs can hinder digestive or metabolic pathways, concurrently adhering to vital nutrients such as zinc, iron, and vitamin E [136].

## 11. Formulations and Delivery Methods

### 11.1. Supplements

Patients with diabetes can regulate their blood sugar levels by consuming foods with a low glycemic index (GI). The findings of Ma et al. (2018) demonstrated that WKB contains an alpha-amylase inhibitor that reduces the estimated GI (eGI) of gluten-rich diets by suppressing the activity of mammalian amylase [137]. The US Food and Drug Administration consider amylase inhibitors from white kidney bean extract under the Generally Recognized As Safe (GRAS) principle. Therefore, interest has grown in the widespread use of white kidney bean extracts for the preparation of low-GI meals [138]. There are still challenges with the industrial techniques of creating low-GI meals [139]. Despite the use of commercial white kidney bean extracts, such as *Phaseolus vulgaris*, to reduce postprandial glucose levels [140]. However, a suitable way to utilize WKBE in the food industry is to consider the variables (such as Temperature, pH, incubation time, etc.) that influence the bean inhibitory activities. Because of its nutritious value and ease of preparation, instant porridge is gaining popularity worldwide [141]. On the other hand, fast porridges’ comparatively high GI is unfavorable to the control of glucose [142]. WKBE suppresses amylase activity and offers a way to lower the GI of different kinds of fast porridge [143]. Research demonstrated that fermented bean-based drinks are manufactured by combining several techniques. They are more nutritious and beneficial to health [144]. The fermentation of bean-based drinks with lactic acid resulted in nutrient-dense nondairy products, compared with those made from dairy. Lactic acid bacteria enhanced the digestibility of fermented beverages by reducing the oligosaccharides that aggregate in the digestive tract. Overall, the fermented-based drinks are suitable for bacterial cells to survive both the fermentation process and the cold storage time. They might be described as functional products that act as probiotic vessels [145].

### 11.2. Fortification

The method of adding an exogenous substance to basic food components to enhance their pro-health properties is termed dietary fortification. Despite this, the model is commonly used to improve both nutritional and nutraceutical qualities of food [145]. A previous study demonstrated that adding phenolic compounds to the food system significantly modified these areas due to the interaction with the food matrix constituents [146]. The interaction between phenolic compounds and the dietary matrix has been reported to negatively influence antioxidant activity and the in vitro bioaccessibility of phenolic compounds [147]. It is feasible to use the combination index to assess the food-matrix interaction as well as the affinity of phenolic compounds for the food matrix. The phenolic-food matrix interaction changed during the simulated digestion, as shown by the comparison of the combination for the sample obtained before and after in vitro digestion. Phenolic compounds typically reduce the digestion of carbohydrates and protein [148]. Moreover, interaction with phenols was demonstrated to modify the electrophoretic and chromatographic profile of the protein. The intricate interaction and the unique attraction of phenolic compounds for food matrix components are considered, since the consequence of phenolic food-matrix interaction depends on the individual phenolic chemical used [149]. In summary, the interaction between phenolic chemicals and the food matrix has a substantial influence on the nutritional potential of fortified foods. Thus, the requirement must be regarded throughout the entire design and assessment process. Nevertheless, due to the complexity of these interactions, food science will continue to face these challenges [150].

### 11.3. Recipes

Most probiotic drinks are naturally made with milk; however, there are plant-based, non-dairy substitutes available. As a result, the beverage samples were produced from germinated WKB seeds using two yoghurt starter cultures [151]. Although the raffinose and stachyose concentrations after fermentation were around 31% and 17% lower, respectively, than before fermentation (2.73 mg kg^−1^ and 0.43 mg kg^−1^, respectively), in contrast, verbascose was not substantially decreased. Riboflavin, pyridoxine, and niacin levels in each sample were decreased significantly after fermentation (around 88%, 45% and 76%, respectively) [152]. In contrast, the levels of thiamine and cyanocobalamin have not changed much. During the 28-day cold storage period, the water holding capacity shifted, ranging from 49 to 57%. Both within the first 28 days of storage at 60 °C and immediately after fermentation, the initial microbe population reached the minimum therapeutic limit. Drinks with a natural flavor were not well received; however, by adding fruit flavors, the acceptability increased by around 15% [153,154].

### 11.4. Cooking Methods

#### 11.4.1. Boiling

Among the most popular techniques for minimizing non-nutritional components in KBs, boiling is a necessary treatment [155]. Wang et al. (2023) [156] documented that both the extent and rate of starch digestion increased principally during boiling of chickpea, cowpea, and white kidney beans, by inactivating trypsin and lectin inhibitors in WKB. Both boiling rate and type of bean had a direct impact on the effectiveness of heat treatment in decreasing these non-nutritional components [156]. In comparison to the whole bean, the phytate content in WKB-based spaghetti reduced from 12.90 to 9.25 mg/g post-boiling. It indicates that milling can potentiate boiling to promote the phytic acid breakdown, which in turn improves the absorption of dietary iron. Similarly, soaking and combined hull processing were observed to increase KB seeds’ relative water absorption, leading to a significant decrease in the tannin and polyphenol content as well as trypsin inhibition in the boiled products [157,158]. Further discovered that the boiling rate influences the phytic acid retention in KBs. Quick cooking varieties, such as red, cranberry, and yellow KBs, retain higher content of phytic acid than the products that are boiled slowly at a medium heat.

#### 11.4.2. Roasting

The raised temperature during roasting can deactivate non-nutritional constituents more quickly than boiling [159]. Sparvoli et al. (2016) [160] documented that roasting reduced the phytic acid content of red KBs by 16.8%, although this level remained stable after the boiling and soaking procedures. During the roasting process, the amount of KB-derived phytic acid may have decreased due to the formation of insoluble compounds with certain minerals [160].

#### 11.4.3. Microwave

The use of different heat treatments can have combined effects. Microwave treatment, like boiling and baking, has been shown to eliminate non-nutritional components from KBs [129]. Jiasheng Wang et al. (2023) highlighted that combining microwave cooking with hot air drying of pre-paste WKB removes gelatinized surface starch, water, lectins, and the firm seed structure, resulting in improved nutrient accessibility and digestibility [161].

## 12. Regulatory Considerations

### 12.1. FDA and Other Regulatory Standards

In the United States, dietary supplements, including those containing white kidney bean extracts, are regulated by the Food and Drug Administration (FDA). FDA regulatory standards mandate accurate labeling and safety for these supplements [162]. Labeling must include essential information on dosage, contraindications, and potential side effects to ensure consumer awareness and transparency. Compliance with these standards is essential for safeguarding public health and maintaining the integrity of these supplements [163].

### 12.2. Safety and GRAS Status

Most animal and human studies indicate that white kidney bean extract (WKBE) is well tolerated, with no adverse effects at commonly administered doses. Subchronic toxicity tests in rats revealed no observed adverse effect levels (NOAEL) up to 2.5–4.0 g/kg/day, indicating a substantial safety margin for human consumption [164]. Human studies involving doses up to 3000 mg/day for several weeks reported no significant alterations in blood parameters, organ function, or adverse effects [85]. However, inadequate cooking may retain harmful lectins and other non-nutritional components [129].

### 12.3. Recommended Dosage Ranges

WKBE dosages ranging from 700 to 1000 mg are commonly employed in clinical studies for weight management. These doses are administered three times daily before meals, totaling 2100–3000 mg/day, for weight management [70,163]. Safety trials establish a maximum daily intake of up to 6–10 g for a 70 kg adult, although most supplements recommend lower doses [165,166]. Furthermore, animal studies corroborate these findings, reporting no adverse effects at substantially higher relative doses [164,167,168,169].

## 13. Conclusions and Future Directions

White kidney beans (*Phaseolus vulgaris*) represent a promising multifunctional nutraceutical could facilitate the development of innovative obesity management strategies, while serving as a primary nutritional source. WKBE acts synergistically with α-amylase inhibitors, dietary fiber, bioactive phytochemicals, and high-quality protein to enhance satiety, lipid homeostasis, glycemic control, and carbohydrate metabolism. Mechanistic studies elucidate additional pathways involving gut microbiota interactions and nutrient bioavailability, while clinical studies validate their function in controlling caloric uptake and reducing postprandial hyperglycemia. The translational efficacy, dose–response relationships, and long-term safety of WKB across inclusive populations, however, are still largely unknown. The full potential of WKBE as an affordable, accessible, and sustainable food source could be achieved through integration into precision nutrition frameworks and sustainable food systems.

To fully harness the potential of white kidney beans, a comprehensive, integrated research agenda is imperative for future investigations. This agenda should commence with rigorous clinical validation via large-scale, long-term randomized controlled trials to definitively establish the safety, dose–response effects, and efficacy of WKB in weight management. Concurrently, food innovation is needed to develop optimized processing and formulation strategies that enhance the bioavailability of active compounds while minimizing antinutritional factors. To advance mechanistic understanding, multi-omics approaches can elucidate precisely how WKB modulates gut microbiota, lipid regulation, and glucose metabolism at the molecular level. Ultimately, this foundational knowledge will enable precision nutrition by facilitating investigations into individual variability in WKB response, predicated on genetic, metabolic, and microbiome profiles, thereby transforming this legume into a potent, personalized public health intervention.

## Figures and Tables

**Table 1:** Clinical Outcomes of WKBE Supplementation.

Trial/Study (Year)	Design	Dose/Duration	Main Outcomes	Efficacy	References
Tzeng et al. (2022)	Clinical/Human	Not specified	Improve insulin sensitivity, reduce fasting glucose, fructosamine and HbA1c.	Promote glycemic control.	[84]
Barrett et al. (2011) Trial 1	11 adults, crossover	1500 mg, single meal	Decrease glucose AUC by 66%, leading to quicker return to baseline.	Dose-dependent effect.	[83]
Barrett et al. (2011) Trial 2	7 adults, crossover	750 mg, single meal	Reduce glucose AUC by 28%.	Lower dose, lower impact.	
Barrett et al. (2011) Trial 3	13 adults, crossover	1500–3000 mg, single meal	(Capsule form) No impact, 3000 mg powder causes hyperglycemia by 34.1%.	Precise formulation is necessary.	
Barrett et al. (2011) Trial 4	13 individuals, FBG > 126 mg/dL.	500 mg coleus/chitosan	Reduced glucose and insulin levels after 30–60 min compared to placebo.	A product developed by combining.	
60-day RCT (n = 101)	Obese adults	1000 mg × 3/day for 60 days	Loss of weight of 1.9 kg, as well as waist circumference.	Placebo: weight loss of 0.4 kg.	[88]
Wang et al. (2020)	Obese adults	35 days, 2400 mg/day	Reduced weight by 2.2 kg, body fat and BMI.	Placebo-controlled.	[85]
84-day RCT (n = 62)	Obese adults	Dosage not specified	3.2 kg weight, 2.8% of fat reduction, 3.7 cm of waist circumference.	Controls are unchanged.	[70]
Jäger et al. (2024)	Double-blind, placebo-controlled	Dosage not specified	Increase fullness, decrease appetite and overindulging and improve portion management.	Modulation of appetite.	
60-day RCT	Obese adults	Dosage not specified	Reduced weight and intake of food enhanced satiety vs. placebo	Consistent with other research findings.	[89]
35-day RCT	Obese adults	2400 mg/day	Lower triglycerides, cholesterol, fat mass, and body weight.	Cardiometabolic advantages.	[85]
84-day RCT (n = 62)	Obese adults	Dosage not specified	Reduction in weight, BMI, waist circumference and improvement in HDL.	Cardiometabolic benefit.	[70]

## Data Availability

The original contributions presented in the study are included in the article, further inquiries can be directed to the corresponding author.
